# Hepatic and cardiac implications of increased toxic amyloid-beta serum level in lipopolysaccharide-induced neuroinflammation in rats: new insights into alleviating therapeutic interventions

**DOI:** 10.1007/s10787-023-01202-3

**Published:** 2023-04-05

**Authors:** Mai M. Anwar, Abeer A. Mabrouk

**Affiliations:** Department of Biochemistry, National Organization for Drug Control and Research (NODCAR)/Egyptian Drug Authority (EDA), Cairo, Egypt

**Keywords:** Alzheimer’s disease, Aβ, Albumin, Cardiac dysfunctions, Hepatic dysfunctions, Neuroinflammation

## Abstract

Neuroinflammation is a devastating predisposing factor for Alzheimer’s disease (AD). A number of clinical findings have reported peripheral disorders among AD patients. Amyloid beta (Aβ) is a toxic physiological aggregate that serves as a triggering factor for hepatic and cardiac disorders related to neurotoxicity. As a drawback of Aβ excessive accumulation in the brain, part of Aβ is believed to readily cross the blood–brain barrier (BBB) into the peripheral circulation resulting in serious inflammatory and toxic cascades acting as a direct bridge to cardiac and hepatic pathophysiology. The main aim is to find out whether neuroinflammation-related AD may result in cardiac and liver dysfunctions. Potential therapeutic interventions are also suggested to alleviate AD’s cardiac and hepatic defects. Male rats were divided into: control group I, lipopolysaccharide (LPS)-neuroinflammatory-induced group II, LPS-neuroinflammatory-induced group treated with sodium hydrogen sulphide donor (NaHS) (group III), and LPS-neuroinflammatory-induced group treated with mesenchymal stem cells (MSCs) (group IV). Behavior and histopathological studies were conducted in addition to the estimation of different biological biomarkers. It was revealed that the increased toxic Aβ level in blood resulted in cardiac and hepatic malfunctions as a drawback of exaggerated inflammatory cascades. The administration of NaHS and MSCs proved their efficiency in combating neuroinflammatory drawbacks by hindering cardiac and hepatic dysfunctions. The consistent direct association of decreased heart and liver functions with increased Aβ levels highlights the direct involvement of AD in other organ complications. Thereby, these findings will open new avenues for combating neuroinflammatory-related AD and long-term asymptomatic toxicity.

**Graphic abstract:**

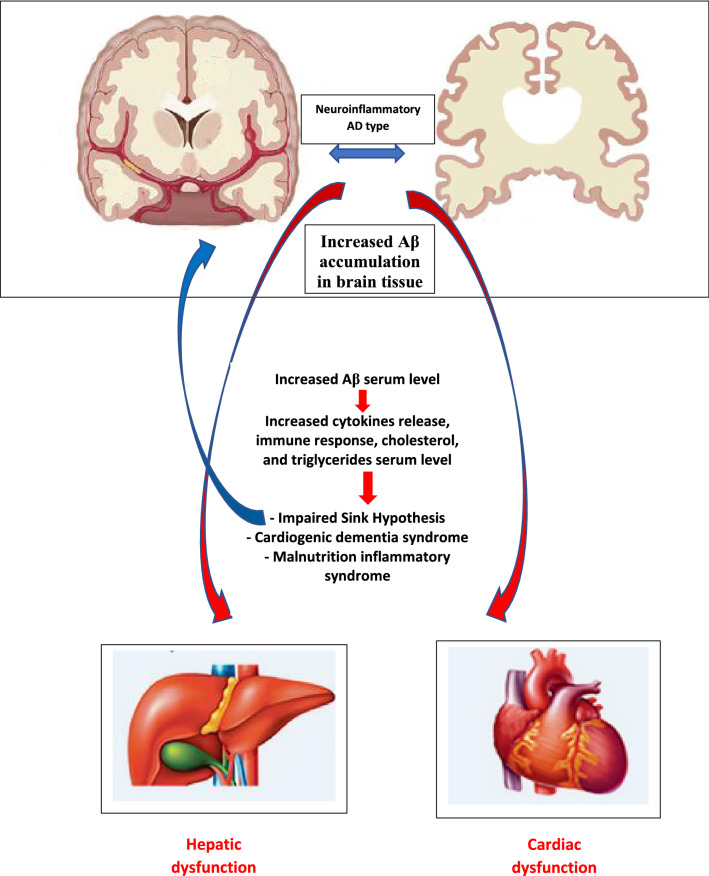

## Introduction

Alzheimer’s disease (AD) is one of the most common types of aging-related neurological disorders that manifests in the form of personal behavior changes, irritability, memory loss, and cognitive dysfunctions (Heneka et al. 2015; Uddin et al. 2022). The pathological drawbacks of AD are related to severe neuronal death that results in a major reduction of the brain’s volume, synaptic transmissions, and metabolic capacity (Toral-Rios et al. 2020; Anwar 2021, 2021a; Xia et al. 2020). Neuroinflammatory AD-associated neuronal death is mainly manifested due to tau and Aβ depositions resulting in an abnormal metabolic system and exaggerated apoptosis. The molecular hypothesis of disease generation is still under intensive investigation (Murphy et al. 2022; Xia et al. 2020). Whereas, several pieces of evidence suggest that age-related chronic inflammation including AD shares repeated risk factors such as environmental, genetic, and molecular mechanisms (Dolan et al. 2010; Casserly and Topol 2004). Aβ peptides are directly generated by specific proteolytic cleavage of the specific amyloid precursor protein (APP) by both beta- and gamma-secretases, respectively (Zhang et al. 2012). It was reported that amyloid-beta aggregations activate successive proinflammatory cascades involving oxidative stress and cytokine release leading to various vascular diseases and organ dysfunctions (Vukic et al. 2009; Thomas et al. 1996; Anwar et al. 2023). AD was previously found to be associated with numerous comorbidities such as cardiovascular diseases and hepatic dysfunctions (Xia et al. 2020; Kay et al. 1987; Stakos et al. 2020). It was observed that the significant increase in AD incidences and progression among the elderly population is associated with other diseases’ sudden onset (Rea et al. 2018; Anwar and Fathi 2022). These findings link and highlight AD’s long-term drawbacks which result in worsening other organs’ normal functions including the heart and liver (Kay et al. 1987; Stakos et al. 2020).

Cerebrovascular disease (CVD) is a severe disease category that centers around impairments arising within the heart and blood vessels reducing heart functional capacity (Francula-Zaninovic and Nola 2018). These impairments manifest in the form of arrhythmia, hypertension, thrombosis, and atherosclerosis (Matheny et al. 2011). CVD has been recently found to coexist as a drawback of AD dysfunctions supporting the idea that the onset of AD intensifies the complications of CVD (Petrovitch et al. 2005; Esiri et al. 2014). Although the influence of this coexisting remains uncertain, understanding the role of AD pathology in early CVD is a vital key to combating cognitive drawbacks supporting the idea that the onset of AD intensifies the complications of CVD. Aβ drainage to the bloodstream is probably the main mechanism of cardiac injury pathogenesis as a drawback of AD progression. Aβ aggregations had led to the hypothesis that these molecules may exert several proinflammatory properties not only cerebrally but also extending to the peripheral and vascular circulation in addition to the aortic and coronary vascular wall leading to progressive heart injury or failure (Koyama et al. 2012; Sullivan et al. 2021).

Metabolic liver activities can be directly correlated with the metabolic readout of the peripheral circulation. Several pre-clinical and clinical studies demonstrated impaired signaling cascades, energy metabolism alterations, and triggered inflammation associated with abnormal liver functions in AD subjects (Clarke et al. 2018; Sookoian and Pirola 2012). These observations agree with the suggestion that AD may act as a risk factor for other organ dysfunctions including the liver. The peripheral blood level of several biological markers such as albumin, alanine aminotransferase (ALT), aspartate aminotransferase (AST), and alkaline phosphatase (ALP) are mainly used to assess liver function and the relevant degree of liver injury (Sookoian and Pirola 2012; Clarke et al. 2018; Yamamoto et al. 2013, 2014; Anwar and Laila 2022). Thereby, these factors are found to be modified upon long-term AD progression as a drawback of increased amyloid β levels in the blood leading to major liver dysfunctions.

Hydrogen sulfide (H_2_S) is a type of novel gaseous endogenous signaling molecule mainly synthesized from L-cysteine by the action of both cystathionine γ-lyase (CSE) and cystathionine β-synthase (CBS) mammalian enzymes tissue types (Yilmaz et al. 2019; Jeddi et al. 2020; Anwar et al. 2019a, b). H_2_S was found to exert potent effects on different types of cells and organs such as smooth muscle cells, endothelial cells, inflammatory cells, brain, heart, liver, and kidney. H_2_S exogenous supplementation attenuates CVD, and hepatic dysfunctions, and neuroinflammation in addition to showing potential anti-inflammatory and antioxidant actions in different animal models (Anwar et al. 2019a, b; Jeddi et al. 2020; Yilmaz et al. 2019). Several studies previously demonstrated that the down expression of the H_2_S-CSE system has a significant role in regulating various diseases such as hypertension, neurodegenerative diseases, and CVD. Administration of H_2_S exogenous donors such as sodium hydrogen sulphide (NaHS) may act as a potent antioxidant, anti-inflammatory, and anti-apoptotic mediator (Anwar et al. 2019a, b; Jeddi et al. 2020; Yilmaz et al. 2019).

Mesenchymal stem cells (MSCs), are adult stem cells type capable of continuous self-renewal and tissue differentiation. They are mainly found in the bone marrow (BM) and can also be identified in several tissues including muscles, adipose tissue, peripheral blood, and the umbilical cord (Hmadcha et al. 2020; Anwar et al. 2021a). When MSCs are cultured under controlled conditions, they can be capable of differentiating into different cells such as cardiomyocytes, neuronal cells hepatocytes, and epithelial cells. These differentiating actions of MSCs proved their potent role in tissue regeneration (Hmadcha et al. 2020; Anwar et al. 2021a). Thus, MSCs have become one of the top most used stem cell types to be used for clinical application due to numerous advantages. MSCs were proven to have the ability to migrate to the injured inflamed organs in response to different triggered immune signaling factors to mediate tissue regeneration potentiated by the release of different pleiotropic effects. MSCs are also capable to inhibit immune system dysregulation and to induce angiogenesis via their pleiotropic activities. All these various advantages of isolated MSCs make this type of cell a powerful regenerative tool for clinical application (Hmadcha et al. 2020; Anwar et al. 2021a).

In this study, we aim to highlight the effect of neuroinflammatory AD type progression on cardiac and liver functions. Thereby, to achieve the goal of illustrating the detailed mechanism of neuroinflammatory-related AD pathogenesis and mechanism of impairments, Lipopolysaccharide (LPS) was directly used to induce AD neuroinflammatory type in male albino rats. Following the induction of AD and hallmarks depositions in the brain, cardiac and hepatic biological parameters were assessed in correlation with Aβ level in the blood. Additionally, a suggested therapeutic intervention using mesenchymal stem cells (MSCs) and exogenous hydrogen sulfide (H_2_S) donor known as sodium hydrogen sulphide (NaHS) were used to find out their hindering effect on cardiac injury and liver dysfunction related to AD.

## Materials and methods

### Chemicals

Lipopolysaccharide (LPS) extracted from Escherichia Coli 0111:B4 and sodium hydrogen sulfide (NaHS) as an exogenous donor type of H_2_S were both directly purchased from Sigma Aldrich (Merck KGaA).

### Preparation, isolation, and analysis of mesenchymal stem cells (MSCs) bone marrow type

**Fig. 1:**
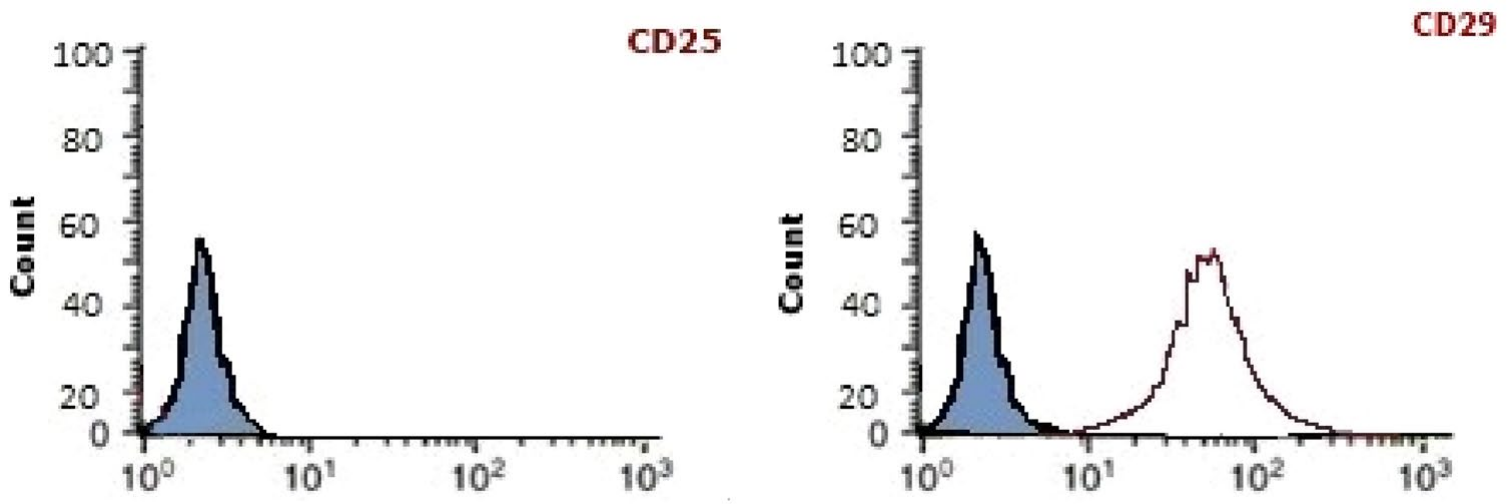
Illustration of bone marrow MSCs type characterization analyses indicating being negative for CD25 and positive for CD29

Initially, bone marrow MSCs were isolated and harvested by careful flushing of the tibiae and femurs of three Albino rats using phosphate-buffered saline (PBS). Bone marrow MSCs were directly placed in a sterilized medium containing the Dulbecco's modified Eagle's medium (DMEM) and 10% fetal bovine serum (FBS). Isolated cells were resuspended in a new culture medium containing 2% penicillin–streptomycin following isolation by density gradient. For 14 days, isolated cells were incubated in a 5% humidified CO_2_ incubator at 37 °C. Obtained large colonies cells were repeatedly washed with PBS supplemented with trypsin (0.25%) for 4–5 min at 37 followed by direct centrifugation at 3000 rpm for 15 min (Anwar et al. 2021b; Abdel Aziz et al. 2007). Obtained first passage bone marrow MSCs cultures were identified by the adherent fusiform structure and using a flow cytometer for detecting CD34-ve, and CD29 + ve as shown in Fig. 1.

### Experimental design

The conducted experimental design was approved by the Animal Research and Ethical committee of the National Organization of Drug Control and Research (NODCAR) approval number (NODCAR/II/57/2022) following the 3Rs main principles (refine, reduce and replace). Twenty-four male Albino rats weighing from 150 to 180 gm (5–6 weeks old) were directly purchased from the animal facility house of (NODCAR, Cairo, Egypt). Rats were housed and kept maintained at a controlled temperature with free access to food and water (22 °C/12-h light/dark cycle). Whereas, the chosen dose for LPS induction assigned for each group of rats was highly considered for affecting the brain only and inducing Aβ hallmarks deposition to avoid confounding effects and to prove out the aims of the current study. The key findings of one of the published research papers proved out that circulating LPS is very rapidly cleared from the systemic circulation, with an approximate half-life of around 2–4 min, and a 5 min clearance rate (80%) following direct infusion (Yao et al. 2016). It is important also to note that the remaining 20% of LPS in the body is potentially involved in neuroinflammation by bounding to immune cells including monocytes, and macrophages (Yao et al. 2016). The 24 male albino rats were divided equally into: control (group I, *n* = 6), LPS-neuroinflammatory-induced rats (group II, *n* = 6): where rats were intraperitoneally (IP) injected with a single dose of LPS (4 mg/kg) dissolved in o.5 ml PBS (Bossù et al. 2012) with certain modifications. The total period of AD induction following LPS injection was 10 days. LPS-neuroinflammatory-induced group treated with NaHS (group III, *n* = 6): where LPS-induced neuroinflammatory rats received NaHS as an exogenous hydrogen sulphide donor (IP) as one type of therapeutic intervention for hepatic and cardiac disorders related to AD at a single daily dose of 5 mg/kg reconstituted with deionized water for 20 days with slight dose modification (Jeddi et al. 2020). Meanwhile, LPS-neuroinflammatory-induced group treated with MSCs (group IV, *n* = 6), where LPS-induced neuroinflammatory rats received a single intravenous dose (I.V) of isolated MSCs 1 μL (500 × 103/μL) as a second therapeutic intervention type for hepatic-cardiac disorders related to AD (Anwar et al. 2021b; Babaei et al. 2012). Following a few hours of LPS injections, observed motion sickness and behavior changes were observed in induced rats as a drawback of neuroinflammatory AD-type induction.

### Morris water maze (MWM) behavior assessment

After 30 days from starting the experimental design and prior to decapitation, all rats were assigned to perform the behavior MWM in order to evaluate the extent of behavior changes as a drawback of neuroinflammatory AD-type induction. All Rats were separately placed in a circular pool filled with opaque water (25 °C). A metal platform was submerged one inch under the opaque water and placed in the middle of the required target quadrant. The training was performed on 5 consecutive days where each trial was meant to not exceed 60 s. A final trial was conducted where the time taken by each rat to reach the submerged platform was directly calculated and recorded (Anwar et al. 2021b).

### Biological assessments

Prior to decapitation, heart rate was measured using Langendorff apparatus (Jeddi et al. 2020). Blood was collected from all rats by retro-orbital puncture followed by centrifugation at 4000 rpm for 15 min to obtain the required serum for further analysis. All rats were sacrificed by decapitation and the brains were rapidly isolated and homogenized according to manufacturing instructions.

### Brain tissue and serum neuroinflammatory AD hallmarks assessment

In order to ensure the induction of neuroinflammatory AD type, Aβ and tau protein were detected in the control and LPS-neuroinflammatory-induced rats using Elisa according to manufacturer instructions (Mybiosource). Meanwhile, Serum Aβ level was detected among all groups. Since the main aim of the conducted study is to highlight the effect of neuroinflammation-related AD progression on the functionality of other organs including the heart and liver, it was vitally important to detect Aβ, and tau protein in LPS-induced neuroinflammatory groups in correlation with other cardiac and hepatic biological parameters. One the other hand, the main goal of the suggested therapeutic intervention represented in the treatment groups was to hinder the neuroinflammatory AD damaging effects on the heart and liver in correlation with Aβ serum level and the degree of hallmarks depositions.

### Cardiac and hepatic biological assessments in serum

To detect the effect of neuroinflammatory AD progression on the heart and liver, several parameters were essentially required to be detected using the colorimetric method including: ammonia (Mybiosourse), lactate dehydrogenase (LDH), Creatine kinase-MB (CK-MB), total protein (TP), albumin, alkaline phosphatase (ALP), Aspartate aminotransferase (AST), Alanine transaminase (ALT) cholesterol and triglycerides (Biomed diagnostics). Meanwhile, troponin I, tumor necrosis factor-alpha (TNF-α), caspase-3, and interleukin 6 (IL-6) were detected using enzyme-linked immunosorbent assays (ELISA) kits purchased from (Mybiosourse) according to manufacturer instructions.

### Histopathological examination and special staining

Brain, heart, and liver were isolated from all groups and were directly fixed in 10% formalin followed by being embedded in paraffin blocks. 3–4 micron thickness were obtained and processed for Hematoxylin and Eosin (H&E) staining to detect histopathological alterations (Ogaly et al. 2015; Schipke et al. 2017). Meanwhile, Masson's trichrome special staining was assessed for heart and liver sections among all groups to detect the degree of necrosis and fibrosis in addition to collagen quantification (Ogaly et al. 2015; Schipke et al. 2017).

### Statistical analysis

Data were expressed as mean ± SD using GraphPad Prism 5.0 and SPSS25 software. The differences among groups were analyzed using one-way ANOVA followed by multiple-group comparisons using the Dunnett’s test. Results were considered significant at *p* < 0.05. Correlations were assessed using the Pearson correlation coefficient. GraphPad Prism 5.0 software was used to draw graphs.

## Results

### Analysis of MSCs cell surface marker expression and flow cytometry characterization

Bone marrow MSCs detection by flow cytometer indicated being negative for CD25-specific marker and positive for CD29 specific marker as shown in Fig. 1.

### Morris water maze behavior test: an assessment of behavior and cognitive dysfunctions

**Fig. 2:**
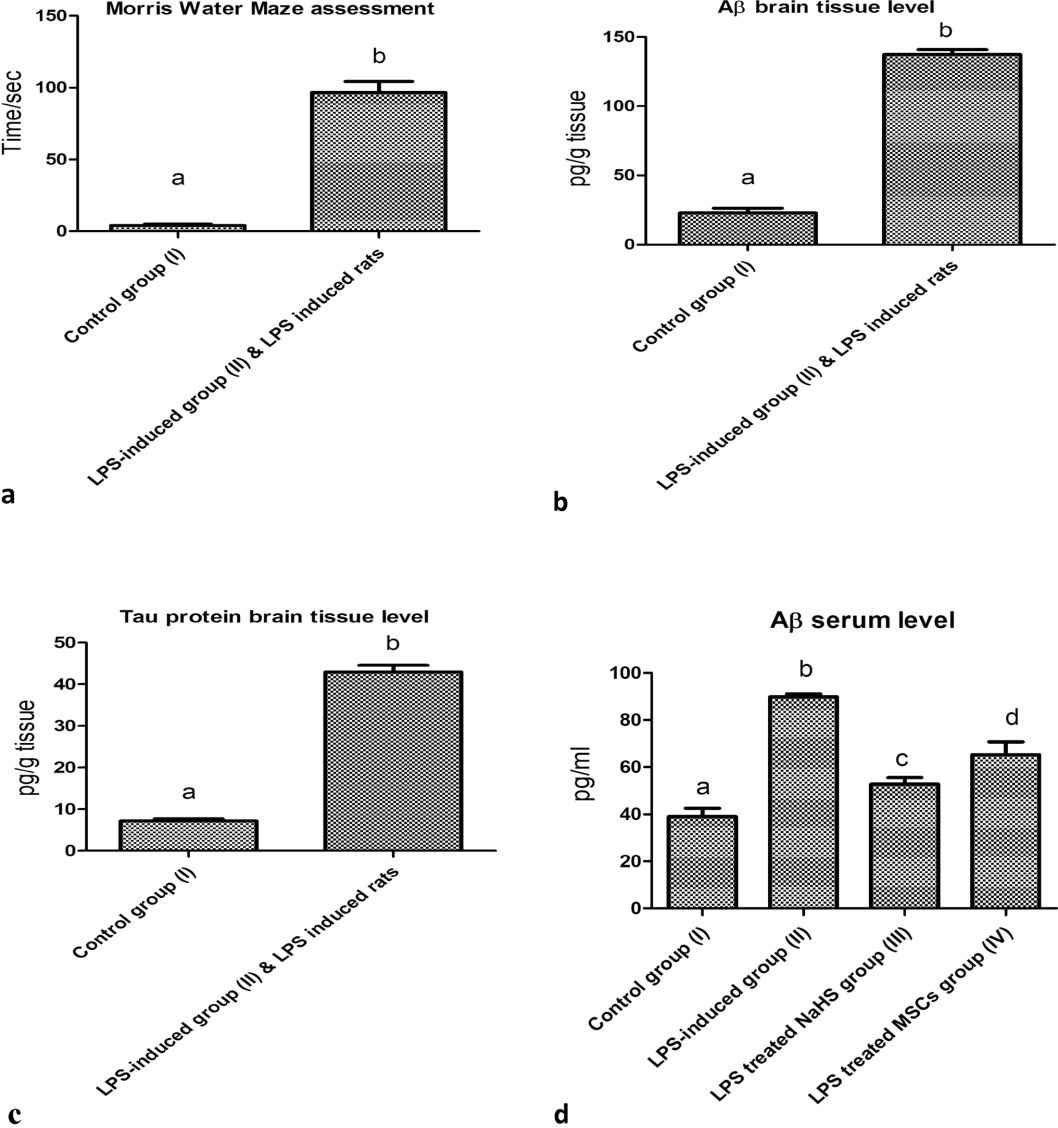
Illustration of neuroinflammatory AD pathological hallmarks and behavior changes. **a** Morris water maze (MWM) behavior assessment comparing LPS-induced neuroinflammatory rats with the control group. **b** Aβ brain tissue level comparing LPS-induced neuroinflammatory rats with the control group. **c** Tau protein brain tissue level comparing LPS-induced neuroinflammatory rats with the control group. **d** Aβ serum level comparing treatment groups (III, IV) with LPS-induced neuroinflammatory group (II), and the control group (I). Results are expressed as Mean ± SD. Different small letters among columns indicates significant difference (*P* < 0.05, *n* = 6)

Subsequently, the current study aims to highlight the drawbacks of neuroinflammatory AD progression in the heart and the liver. AD inflammatory type was initially induced in rats by a single interpretational injection of LPS. MWM was assessed to indicate the complete induction of neuroinflammatory AD type in order to study the deteriorative effects of neuroinflammation on the heart and liver. Our results indicated that the LPS-induced neuroinflammatory rats significantly consumed the longest time in MWM when compared with the control group (I) (*P* < 0.05, *n* = 6) as shown in Fig. 2(a).

### Relevant Aβ and tau hallmarks deposition in brain tissues in correlation with neuroinflammatory AD progression

Aβ and tau protein hallmarks deposition has been assigned in rat’s brain tissue to assure the complete induction of AD inflammatory type. Significant increase in Aβ and tau brain tissues level was observed in neuroinflammatory AD rats when compared with the control group (I) (*P* < 0.05, *n* = 6) as shown in Fig. 2(b,c).

### Increased amyloid β serum level as the main predisposing factor for cardiac and hepatic dysfunctions related to neuroinflammatory AD type progression

A significant increase in Aβ serum level was observed following the induction of neuroinflammatory AD type in group (II) when compared with the control group (I) (*P* < 0.05, *n* = 6). A relevant decrease in Aβ serum level was observed in LPS-induced neuroinflammatory-treated MSCs group (IV) when compared with both control and AD group II (*P* < 0.05, *n* = 6). Meanwhile, the most significant effect on Aβ serum level was observed following the daily single administration of NaHS as hydrogen sulphide exogenous donor when compared with both control group (I) and LPS-induced neuroinflammatory group (II) respectively (*P* < 0.05, *n* = 6) as shown in Fig. 2(d).

### Elevated cholesterol and triglycerides serum levels as a drawback of neuroinflammatory AD type progression

**Fig. 3:**
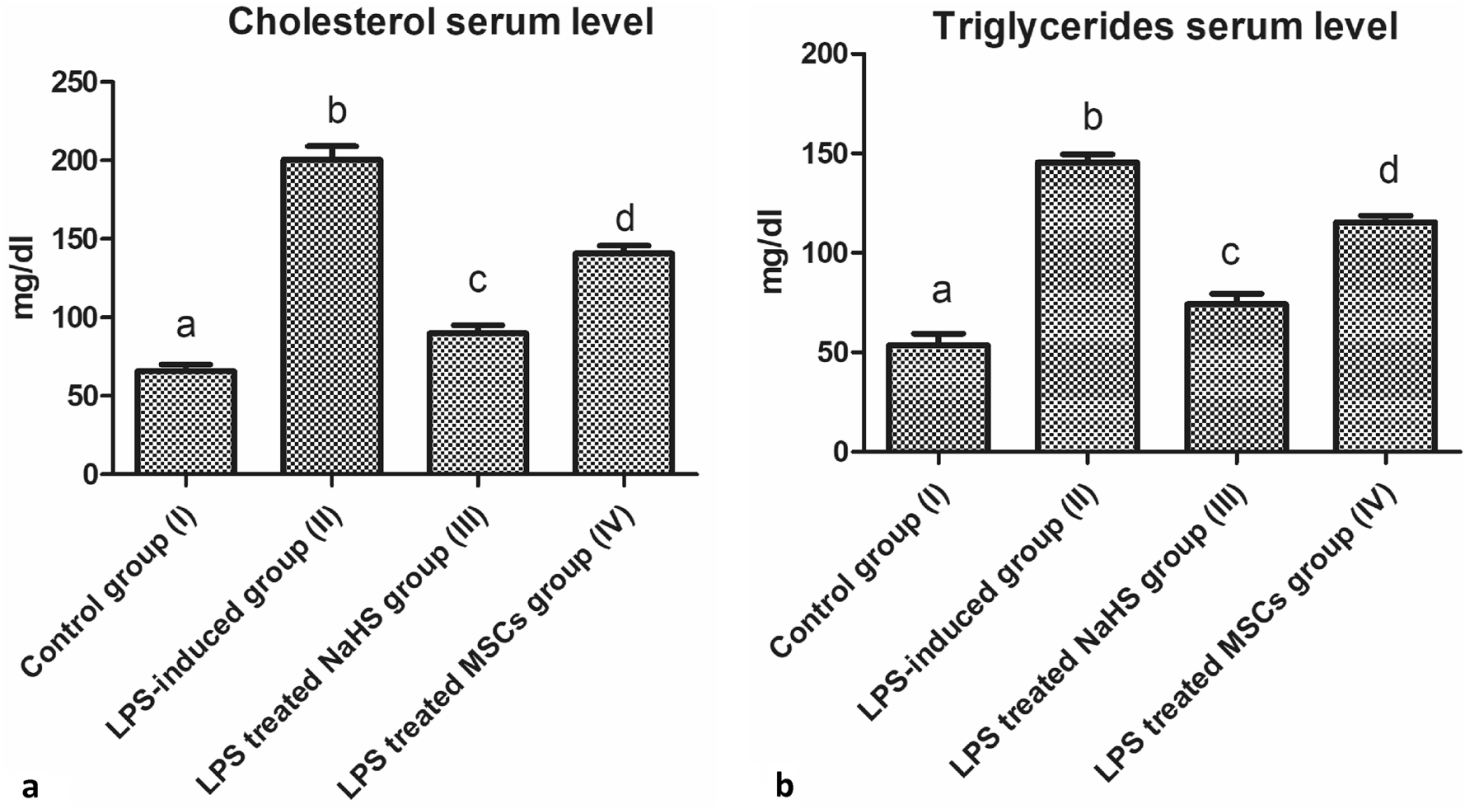
Illustration of cholesterol and triglycerides elevated serum level related to neuroinflammatory AD type progression. **a** Cholesterol serum level assessment comparing treatment groups (III, IV) with LPS-induced neuroinflammatory group (II) and the control group (I). **b** Cholesterol serum level assessment comparing treatment groups (III, IV) with LPS-induced neuroinflammatory group (II) and the control group (I). Results are expressed as Mean ± SD. Different small letters among columns indicates significant difference (*P* < 0.05, *n* = 6)

Our results revealed that following Aβ serum level elevation in LPS-induced neuroinflammatory group (II), a significant increase in both cholesterol and triglycerides serum levels was observed as shown in Fig. 3(a,b) when compared with the control group (I) (*P* < 0.05, *n* = 6). The administration of MSCs to LPS-induced neuroinflammatory rats group (IV) resulted in a marked decrease in both cholesterol and triglycerides serum levels when compared with both control group (I) and LPS-induced neuroinflammatory group (II) (*P* < 0.05, *n* = 6). A significant and remarkable attenuating effect on cholesterol and triglycerides levels was noticed following the administration of NaHS to the LPS-induced neuroinflammatory rats group (III) when compared with both control group (I) and LPS-induced neuroinflammatory group (II) (*P* < 0.05, *n* = 6) as shown in Fig. 3(a,b).

### Triggered inflammatory factors as a drawback of neuroinflammatory AD type progression

**Fig. 4:**
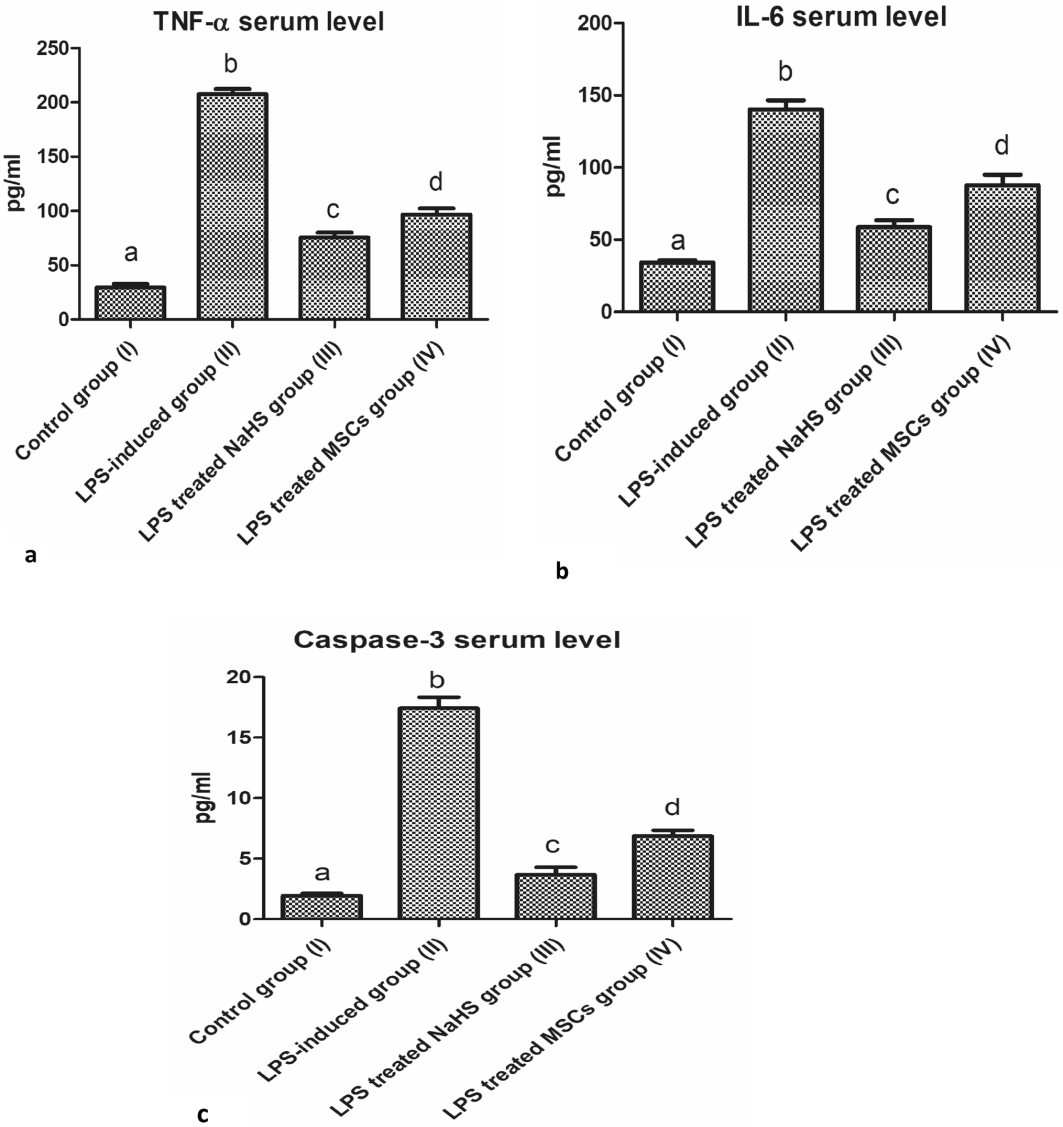
Illustration of inflammatory factors exaggerated release as a drawback of elevated Aβ serum level. **a** Elevated TNF-α serum level comparing treatment groups (III, IV) with LPS-induced neuroinflammatory group (II), and the control group (I). **b** Elevated IL-6 serum level comparing treatment groups (III, IV) with LPS-induced neuroinflammatory group (II), and the control group (I). **c** Elevated caspase-3 serum level comparing treatment groups (III, IV) with LPS-induced neuroinflammatory group (II), and the control group (I). Results are expressed as Mean ± SD. Different small letters among columns indicates significant difference (*P* < 0.05, *n* = 6)

The significant increase in Aβ serum level was displayed and found to be associated with a well-marked increase in TNF-α, IL-6, and caspase-3 release levels following the induction of neuroinflammatory AD type in group (II) when compared with the control group (I) (*P* < 0.05, *n* = 6) as shown in Fig. 4(a–c). The single administration of NaHS and MSCs revealed a significant decrease in TNF-α, IL-6, and caspase-3 levels with a more remarkable decrease following the administration of NaHS when compared with both control group (I) and LPS-induced neuroinflammatory group (II) (*P* < 0.05, *n* = 6) as shown in Fig. 4(a–c).

### Assessment of cardiac injury as a drawback of AD and the effect of therapeutic interventions on alleviating neuroinflammatory AD-related defects

**Fig. 5:**
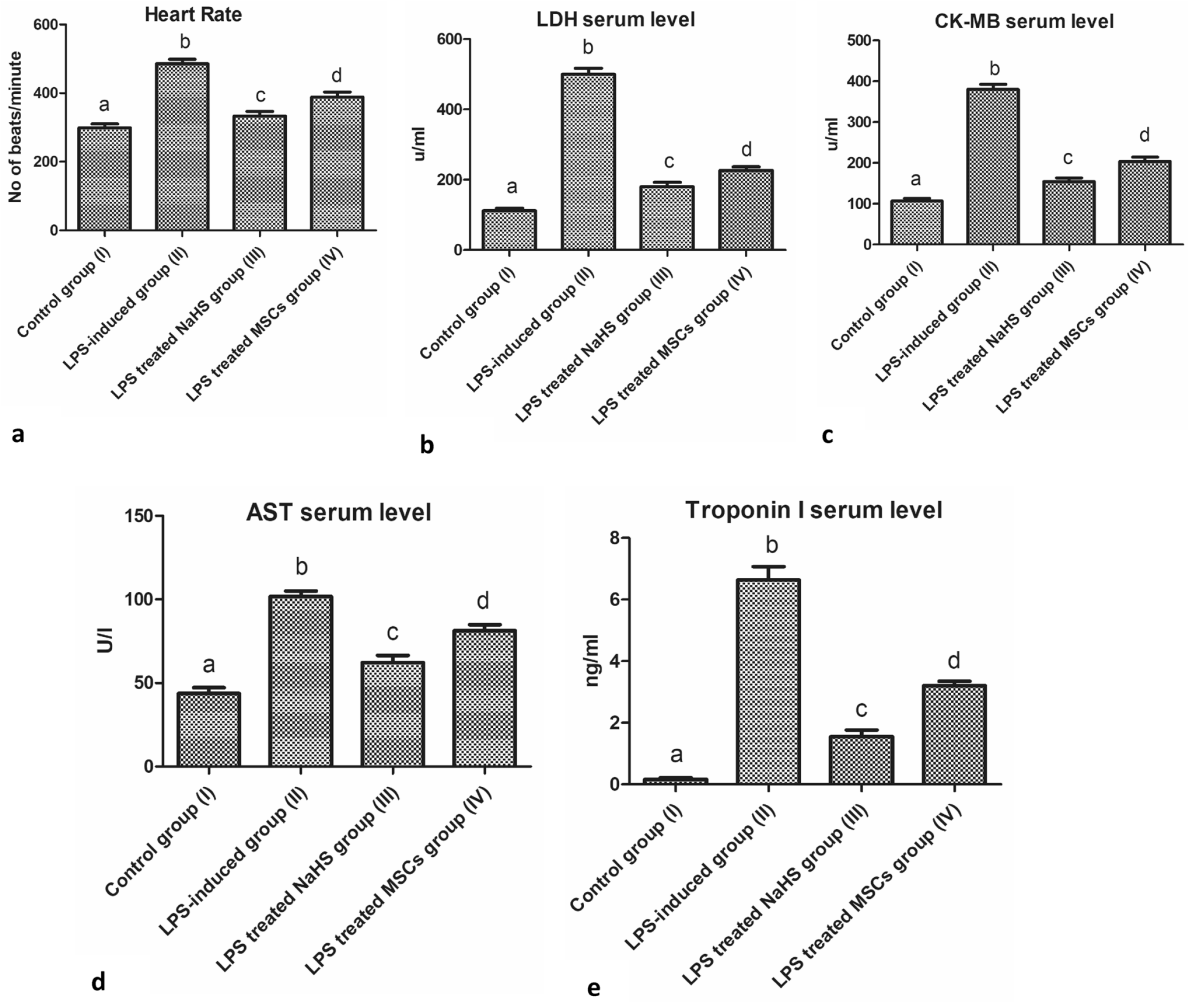
Illustration of cardiac dysfunctions related to AD progression and the efficiency of therapeutic interventions on alleviating AD-related defects. **a** Increased HR of the neuroinflammatory-induced rats and the alleviating effects of therapeutic intervention comparing treatment groups (III, IV) with the LPS-induced neuroinflammatory group (II), and the control group (I). **b** Increased LDH of the neuroinflammatory-induced rats and the alleviating effects of therapeutic intervention comparing treatment groups (III, IV) with the LPS-induced neuroinflammatory group (II) and the control group (I). **c** Increased CK-MB of the neuroinflammatory-induced rats and the alleviating effects of therapeutic intervention comparing treatment groups (III, IV) with the LPS-induced neuroinflammatory group (II), and the control group (I). **d** Increased AST of the neuroinflammatory-induced rats and the alleviating effects of therapeutic intervention comparing treatment groups (III, IV) with the LPS-induced neuroinflammatory group (II), and the control group (I). **e** Increased Troponin I of the neuroinflammatory-induced rats and the alleviating effects of therapeutic intervention comparing treatment groups (III, IV) with the LPS-induced neuroinflammatory group (II), and the control group (I). Results are expressed as Mean ± SD (*n* = 6 rat per group). Different small letters among columns indicates significant difference (*P* < 0.05, *n* = 6)

Upon neuroinflammatory AD-related pathology which resulted in increased Aβ serum level, elevated lipid profile, triggered inflammatory factors release, and disturbance in cardiac functionality have been observed in LPS-induced neuroinflammatory rats group (II) when compared with the control group (I). Thereby, a relevant observed increase in heart rate (HR), LDH, AST, CK-MB, and troponin I levels has been detected in the LPS-induced neuroinflammatory group (II) when compared with the control group (I) (*P* < 0.05, *n* = 6) as shown in Fig. 5(a–e). Following the administration of our suggested therapeutic intervention NaHS (group (III) and MSCs group (IV), a significant observed decrease in HR, LDH, AST, CK-MB, and troponin I levels was recognized among treated groups (III, IV) with a more relevant decrease following the administration of NaHS group (III) when compared with both control group (I) and LPS-induced neuroinflammatory group (II) (*P* < 0.05, *n* = 6) as shown in Fig. 5 (a–e).

### Hepatic deteriorative effects related to AD Progression and the efficiency of therapeutic interventions in alleviating neuroinflammatory AD-related defects

**Fig. 6:**
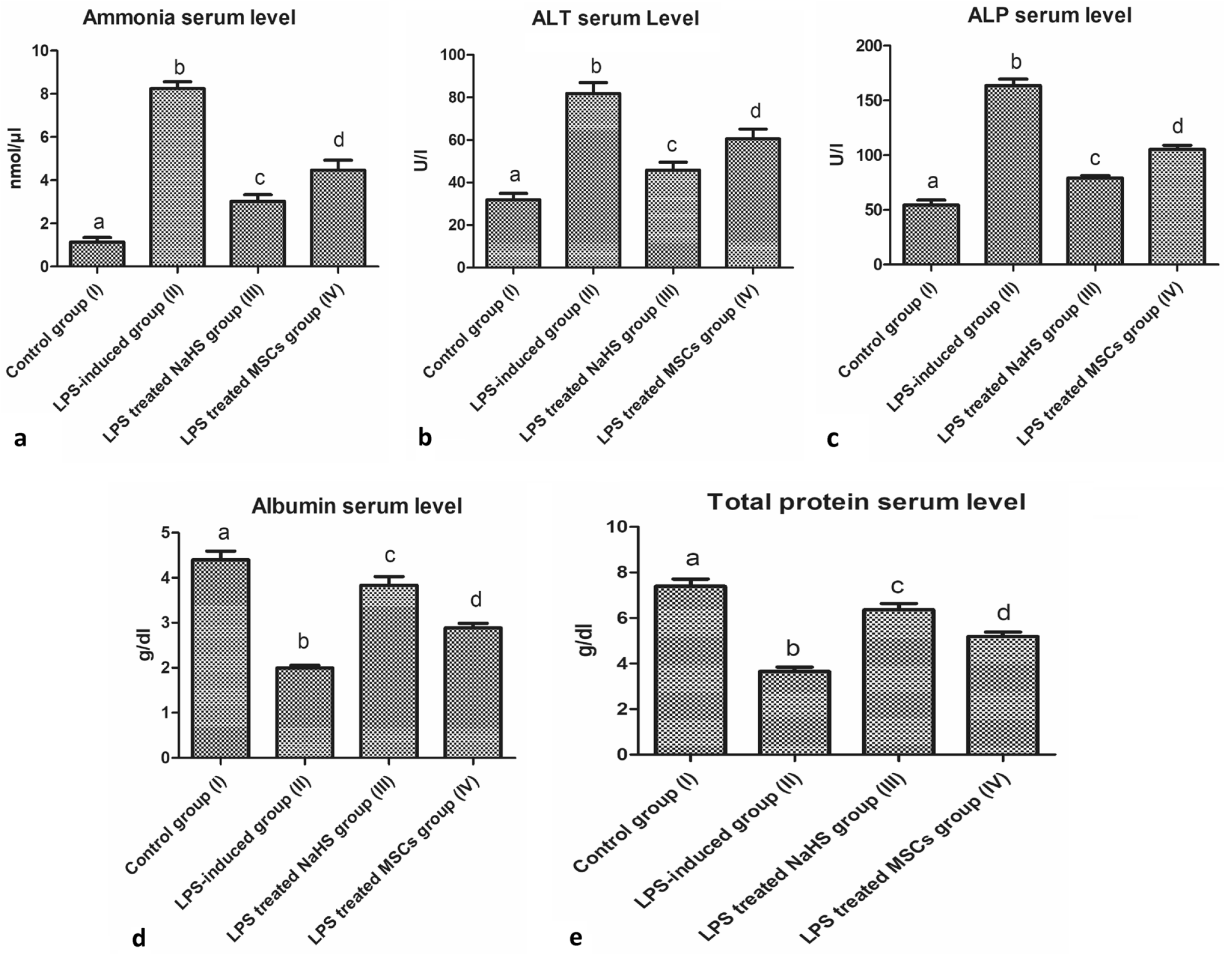
Deteriorative effects of AD progression on liver functionality and the efficiency of therapeutic interventions on alleviating AD-related defects. **a** Increased ammonia serum level of the neuroinflammatory-induced rats and the alleviating effects of the therapeutic intervention comparing treatment groups (III, IV) with the LPS-induced neuroinflammatory group (II), and the control group (I). **b** Increased ALT serum level of the neuroinflammatory-induced rats and the alleviating effects of the therapeutic intervention comparing treatment groups (III, IV) with the LPS-induced neuroinflammatory group (II), and the control group (I). **c** Increased ALP serum level of the neuroinflammatory-induced rats and the alleviating effects of therapeutic intervention comparing treatment groups (III, IV) with the LPS-induced neuroinflammatory group (II), and the control group (I). **d** Decreased albumin serum level of the neuroinflammatory-induced rats and the alleviating effects of therapeutic intervention comparing the treatment groups (III, IV) with the LPS-induced neuroinflammatory group (II) and the control group (I). **e** Decreased total protein of the neuroinflammatory-induced rats and the alleviating effects of therapeutic intervention comparing treatment groups (III, IV) with the LPS-induced neuroinflammatory group (II), and the control group (I). Results are expressed as Mean ± SD (*n* = 6 rat per group). Different small letters among columns indicates significant difference (*P* < 0.05, *n* = 6)

Significant hepatic dysfunctions have been observed in LPS-induced neuroinflammatory rats expressed in the form of increased ammonia, AST, ALT, and alkaline phosphates serum levels with a marked decrease in albumin, and TP serum levels when compared with the control group (I). Meanwhile, the administration of a single dose of NaHS as an exogenous H_2_S donor effectively resulted in very relevant improvement in liver estimated parameters when compared with control group (I) (*P* < 0.05, *n* = 6) as shown in Fig. 6(a–e). While the administration of a single dose of MSCs effectively alleviated the neuroinflammatory AD-induced hepatic dysfunctions when compared with the control group (I) (*P* < 0.05, *n* = 6) as shown in Fig. 6 (a,b,c,d,e) but not as relevant as NaHS group (III).

### Correlation study

**Fig. 7:**
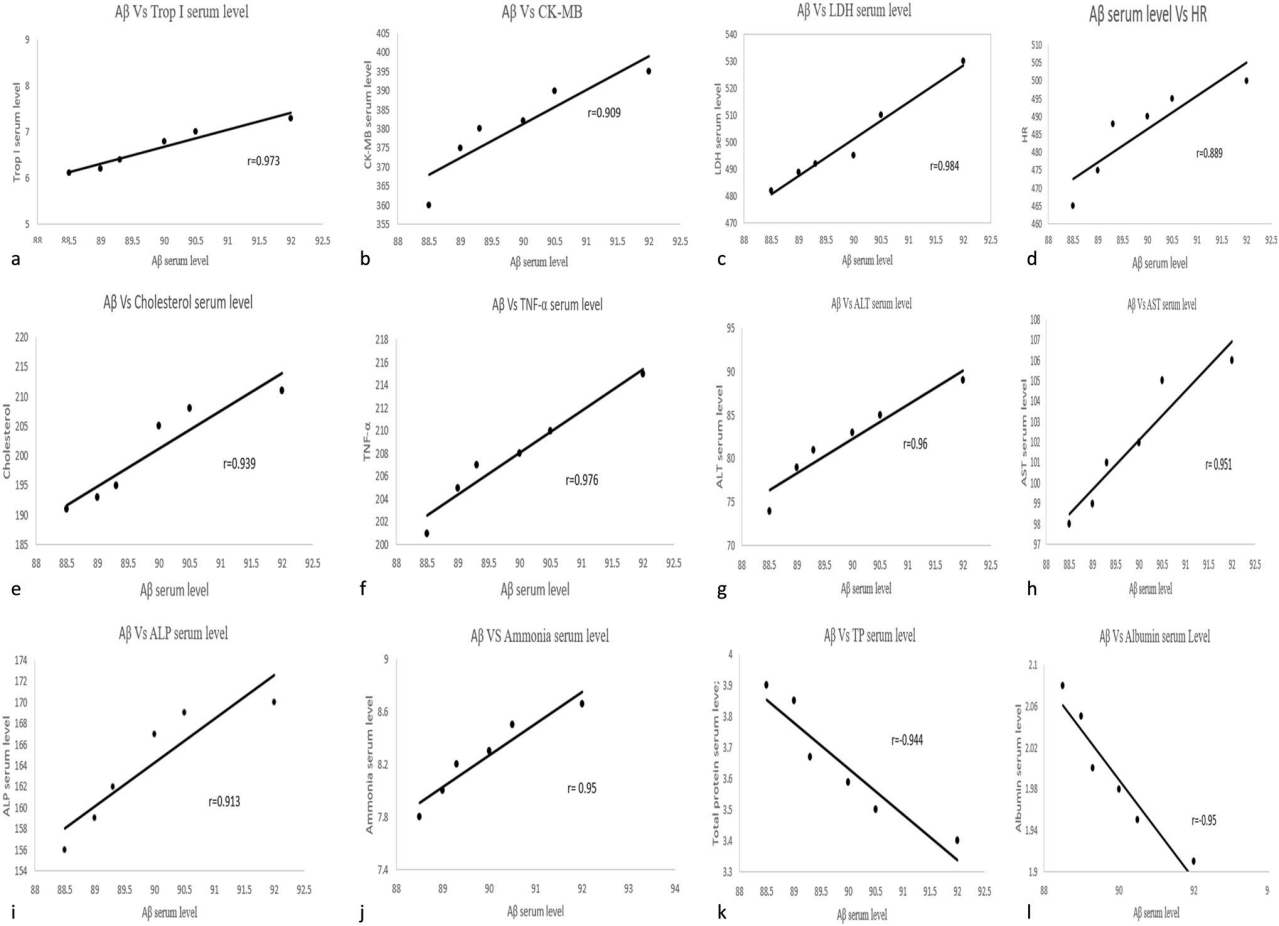
Pearson’s correlation illustration of **a** Aβ Vs Trop I serum level, **b** Aβ Vs CK-MB, **c** Aβ Vs LDH serum level, **d** Aβ serum level Vs HR, **e** Aβ Vs Cholesterol serum level, **f** Aβ Vs TNF-α serum level, **g** Aβ Vs ALT serum level, **h** Aβ Vs AST serum level, **i** Aβ Vs ALP serum level **j** Aβ VS Ammonia serum level, **k** Aβ Vs TP serum level, and **l** Aβ Vs Albumin serum Level

A correlation study was assessed to highlight the extent of the degree of significance, variability, and direct relationship between different tested variables. It was observed a significant positive correlation between Aβ Vs Trop I serum level, Aβ Vs CK-MB, Aβ Vs LDH serum level, Aβ serum level Vs HR, Aβ Vs Cholesterol serum level, Aβ Vs TNF-α serum level, Aβ Vs ALT serum level, Aβ Vs AST serum level, Aβ Vs ALP serum level, and Aβ VS Ammonia serum level as shown in Fig. 7(a–j), respectively. Meanwhile, a negative correlation was observed between Aβ Vs TP serum level and Aβ Vs Albumin serum Level as shown in Fig. 7(k,l). Pearson correlation analysis showed a significant positive and negative correlation among selected parameters indicating the drawbacks of neuroinflammatory AD type progression on selected parameters.

### Histopathological findings of neuroinflammatory AD type-induced cardiac injury with alleviating effects following therapeutic interventions

**Fig. 8:**
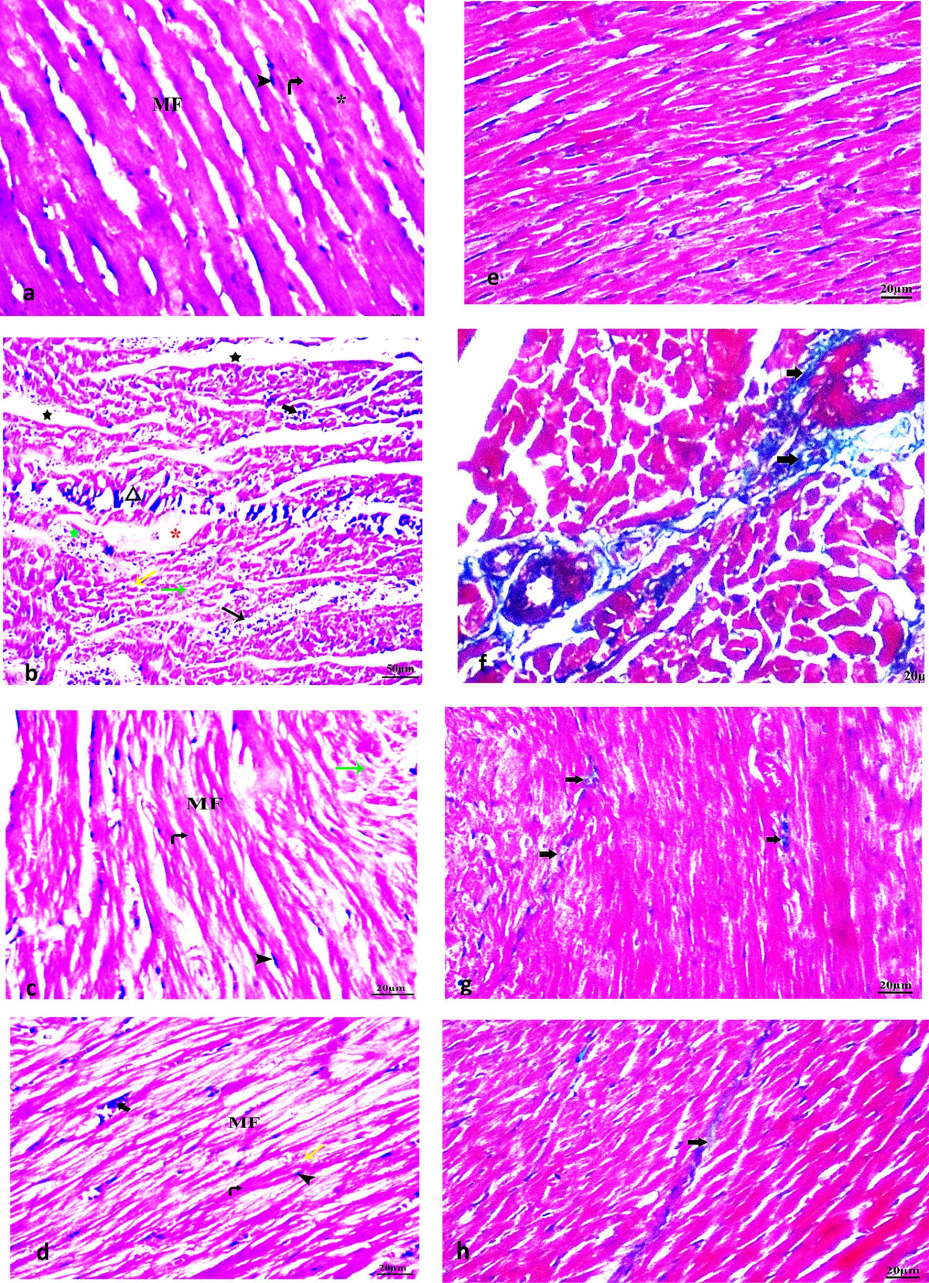
Illustration of histopathological examination of cardiac tissues among subjected groups. Control, LPS-induced neuroinflammatory, NaHS-treated group, and MSCs-treated group, respectively (**a**–**d**) and Masson's trichrome special staining (**e**–**h**). Abbreviations: Acidophilic sarcoplasm (curved arrows), vesicular nuclei (*), blood capillaries (thin arrow), cytoplasmic vacuoles (yellow arrow), interstitial cells with elongated nuclei (arrow heads)Interstitial edema (star), dilated and congested blood vessels (long arrow), hemorrhage (red asterisk), inflammatory cellular infiltration (short arrow), cytolysis of myocytes (green arrows), areas of discontinuation (triangle), myocytes represent disintegrated nuclei (wavy arrow), muscle fibers (MF), thick fibers (K), thin fibers (T), necrosis (green star), hyperacidophilic cytoplasm and pyknotic nuclei (bifid arrow)

Our observed results revealed that the control group (I) showed normal branching and anastomosing longitudinal muscle fibers, clear acidophilic sarcoplasm, central oval vesicular nuclei of the cardiac myocytes, and normal cardiac interstitial cells with elongated nuclei as shown in Fig. 8(a). On the other hand, LPS-induced neuroinflammatory group (II) revealed a misrepresentation of cardiac muscle striation. A noticeable increase in the wide spaces located between the cardiomyocytes, dilated and congested blood vessels, intrafibrillar hemorrhage, and severe inflammatory cellular infiltration was relevantly observed. Additionally, focal areas of impairment and cytolysis of myocytes, loss of sarcoplasmic striations of the cardiomyocytes with areas of discontinuation, variable size of the muscle fibers, degeneration and severe necrosis associated with noticeable apoptotic muscle fibers, major hyperacidophilic cytoplasm, and pyknotic nuclei were also detected as shown in Fig. 8(b). Meanwhile, LPS-induced neuroinflammatory-treated NaHS group (III) revealed preserved cardiomyocytes morphology with central oval vesicular nuclei, minor disintegrated nuclei, and cytoplasmic vacuoles. Almost regular tissue space with minor dilated blood vessels and cellular infiltration were detected with no hemorrhage areas as shown in Fig. 8(c). Meanwhile, LPS-induced neuroinflammatory-treated MSCs group (IV) showed closely adjacent and regular longitudinal cardiac muscle fibers with central oval nuclei. Noticeable focal spaces between myocytes and little count of congested blood vessels surrounded by mononuclear cellular infiltration were detected without any hemorrhage area as shown in Fig. 8(d).

Masson's trichrome heart staining was conducted among all the groups to assess the degree of collagen fiber distribution within cardiac areas. Control group (I) showed normal limited bluish color around the blood vessels and was also found to be faint between the heart muscle cells as shown in Fig. 8(e). On the other hand, the LPS-induced neuroinflammatory group (II) showed excessive collagen accumulation between cardiomyocytes. Relevant scattered connective tissues between cardiac muscle cells with thickened blood vessel walls were also observed when compared with the control group (I) as shown in Fig. 8(f). The administration of NaHS group (III) and MSCs group (IV) resulted in a significant decrease in collagen depositions with only a few thin blue fibers visible surrounding the blood vessels as shown in Fig. 8(g,h). A more relevant decrease in collagen accumulation was observed following NaHS than MSCs when compared with the control group (I) and LPS-induced neuroinflammatory group (II) as shown in Fig. 8(g,h).

### Histopathological findings of neuroinflammatory AD type-related hepatic injury with alleviating effects following therapeutic interventions

**Fig. 9:**
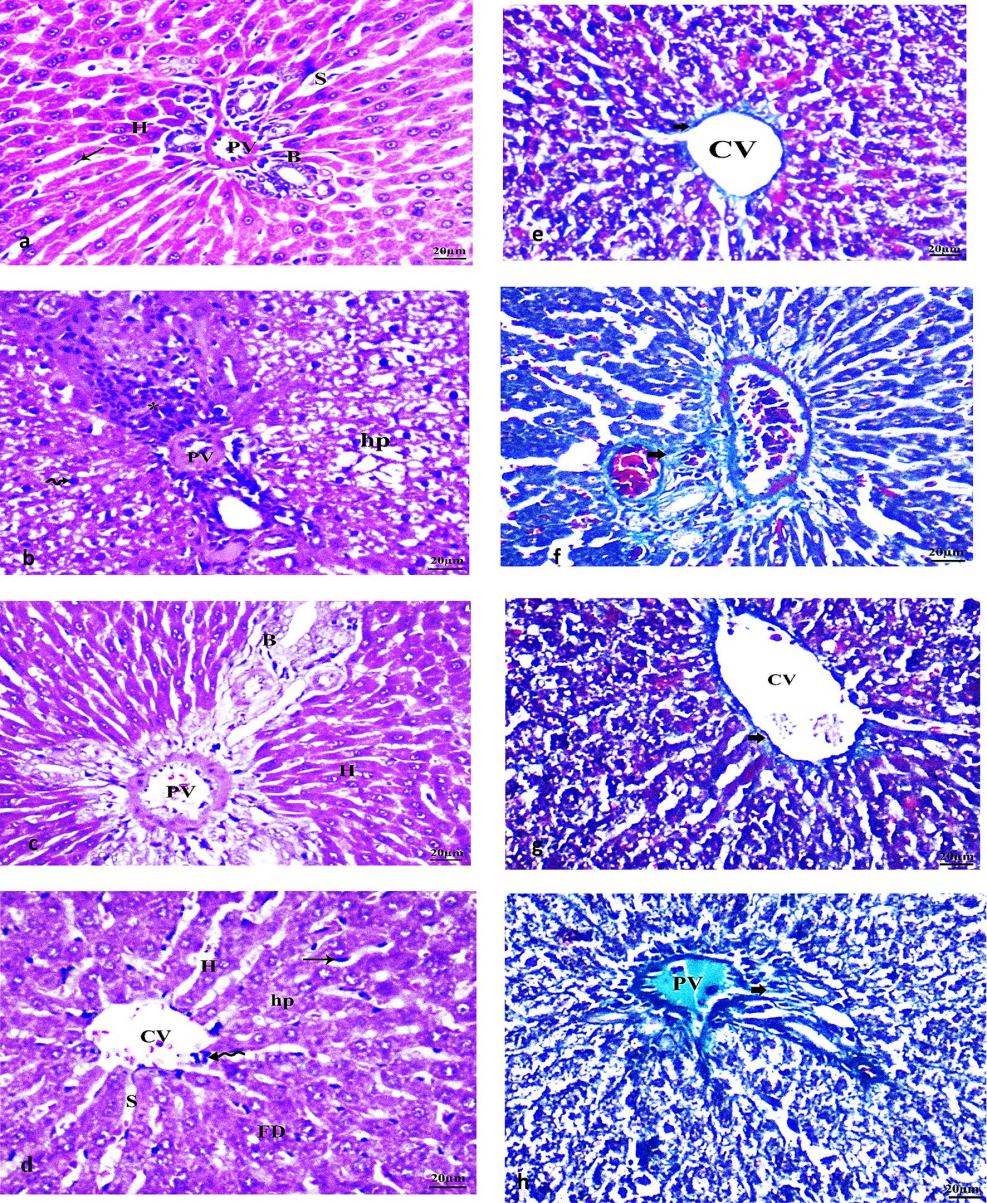
Illustration of histopathological examination of hepatic tissues among subjected groups. Control, LPS-induced neuroinflammatory, NaHS-treated group, and MSCs-treated group, respectively (**a**–**d**) and Masson's trichrome special staining (**e**–**h**). Abbreviations: *H*  hepatic cell or hepatocyte, *S*  sinusoid, *CV*  central Vein, *PV*  portal vein, *B*  bile duct, *Star*  necrotic area with mononuclear cells invasion, *Wavy arrow*  pyknotic nuclei, *FD*  fatty degeneration, *portal triad invaded by lymphocytes, *hp*  degenerated hepatocytes,  = hydropic degeneration, *Zigzag*  lymphatic infiltration, *thin arrow*  kupffer cell, *bold arrow*  karyolysis, and *curved arrow*  cytoplasmic vacuoles

Our observed results revealed that the control group (I) showed no histological deformities, as well as a distinctive central vein and hepatocyte organization. Normal Hepatic strands that travel from the lobule's border to the central vein, besides sinusoids with the portal region were observed without any related damage as shown in Fig. 9(a). Meanwhile, LPS-induced neuroinflammatory group (II) revealed hydropic deteriorating cells, altered lobular form, nuclear degradation in certain regions, disarranged hepatic cells, necrosis, fatty degeneration, and darkly stained nuclei of some hepatocytes (pyknosis) were all observed as shown in Fig. 9(b). Additionally, the hepatic central vein became enlarged and congested. The portal area was found to have leucocytes penetration along with hepatocytes with cytoplasmic vacuoles as shown in Fig. 9(b). on the other hand, both treated groups including NaHS and MSCs showed significant progression in the state of hepatic damage, fibrosis, and injuries as shown in Fig. 9(c,d) with a more relevant alleviating effects following the administration of NaHS as shown in Fig. 9(c,d).

Collagen assessment using Masson's trichrome special staining revealed normal collagen fibers surrounding blood vessels in the central region among control group (I) (Blue color was used to stain collagen (arrow)) as shown in Fig. 9(e). Meanwhile, LPS-induced neuroinflammatory group (II) significantly demonstrated collagen deposition detected in the portal areas and surrounding the central vein in addition to the formation of thick septa between hepatic cells (arrow) as shown in Fig. 9(f). Collagen depositions were significantly reduced with minor thin blue fibers (arrow) detected around the blood vessels in LPS-induced neuroinflammatory-treated NaHS group as shown in Fig. 9(g). Correctly arranged hepatic cords around sinusoids with normal visual hepatic appearance were also detected with slight congestion, pyknosis/necrotic alterations, and mononuclear cell invasion without any fatty degeneration as shown in Fig. 9(g). While the LPS-induced neuroinflammatory-treated MSCs’ group (IV) revealed a minor reduced proliferation of collagen fibers with the presence of blue thin fibrous strands (arrow) extended nearby portal vessels as shown in Fig. 9(h). Minor inflammatory cell infiltrations neighboring the portal triads in hepatic sections were also observed with hepatocytes degeneration and darkly pigmented nuclei, minor fatty deterioration, and moderate central-portal veins congestion as shown in Fig. 9(h).

## Discussion

Lately, the number of neuroinflammatory AD subjects has been noticed to be rapidly increasing over the past few years. As a consequence, cognitive impairment and neuroinflammation have become huge medical and social problems associated with several drawbacks including other organs disorders and altered normal vital functions. Unlike other diseases, the correlation between neuroinflammatory AD type and heart-liver abnormalities remains undefined and unclear. Although several studies have previously reported the associated risk of cardiac and liver dysfunctions to AD, up to now a detailed mechanism of these associated risks and drawbacks has been scarcely explained. A Turkish study previously reported that 29 AD patients suffered from diastolic and cardiac dysfunctions when compared with normal control patients (Çalık et al. 2014). Meanwhile, other two studies reported arterial stiffness and heart failure in several AD patients (Hanon et al. 2005; Zuccalà et al. 2005b). Additionally, other inpatient clinical studies reported liver dysfunctions associated with low serum albumin levels as a drawback of long-term AD when compared with control patients (Zuccalà et al. 2005b; Cattin et al. 1997b; Kim et al. 2020). Since limited valid information is available on the drawback of AD progression on other organs such as the heart and liver. The main aim of the current study is to achieve a better knowledge of the other diseases and factors associated with neuroinflammatory AD type to improve the quality of life for AD patients along with suggesting a therapeutic intervention for combating neuroinflammatory AD type-associated diseases including heart and liver dysfunctions.

Neurodegenerative diseases especially AD have been proven to be driven by neuroinflammation as a drawback of serious inflammatory cascades. These inflammatory cascades in the long term mainly result in AD neuropathological hallmarks accumulation including extracellular amyloid-beta (Aβ) and hyperphosphorylated tau protein. Based on that, the AD neuroinflammatory model was used in the current study in order to study the drawbacks of AD on the heart and liver by using lipopolysaccharide (LPS) as an AD inducer in rats. LPS is a potent endotoxin component of Gram-negative bacteria. It stimulates the release of proinflammatory cytokines resulting in neuroinflammation and ending with AD. Following ten days of IP LPS injection, behavioral stress known as sickness behavior represented in the form of fever, decreased food and water consumption, limited interactions, and reduced exploration was observed among AD rats in MWM (Anwar et al. 2018). The induction of AD results in more consumed time in MWM when compared with control group in correlation with elevated Aβ and tau protein brain tissue and serum levels. Regarding the suggested possible  pathological mechanism indicating the direct link between neuroinflammatory AD type and cardiac-hepatic dysfunctions, our observed results justify and correlates the hypothesis that the elevated Aβ serum level act as the driven triggered factor for neuroinflammatory AD type-related organ dysfunctions.

**Fig. 10:**
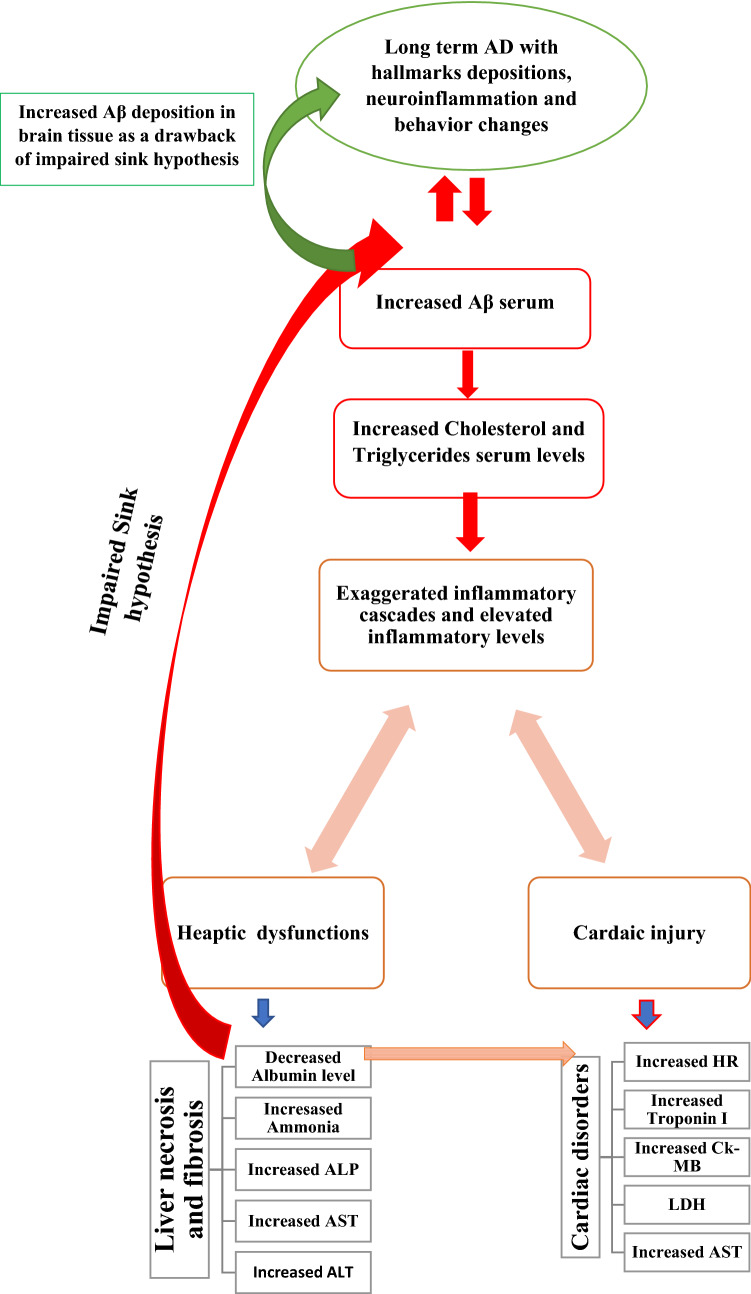
Illustration of amyloid-beta serum level as a triggering factor for cardiac and hepatic dysfunctions as a drawback of neuroinflammatory AD type progression

Intriguingly, the issue of cardiac and hepatic dysfunctions related to neuroinflammatory AD type including cardiogenic dementia syndrome, and the hepatic sink hypothesis is suggested to be triggered and be a drawback of Aβ inflammatory complex syndrome (McCarthy et al. 1981; Kalantar-Zadeh et al. 2004; DeMattos et al. 2002; Boada et al. 2009). Our results revealed that following the increase of amyloid-beta and tau protein levels in brain tissue associated with observed behavior changes in LPS-induced neuroinflammatory rats, serious of systematic and localized damage has been observed among LPS-induced group when compared with the control group (I) (Hardy and Higgins 1992; Koyama et al. 2012). In agreement with our results, the amount of Aβ depositions in brain tissue reflects the degree of AD clinical expression along with the degree of other organ dysfunctions and damage (Moreno-Treviño et al. 2015; de la Torre 2018). Even though aggregated Aβ protein was supposed to be substantially degraded in the brain, a significant amount was found to be transported into the peripheral circulation across the blood-brain barrier resulting in elevated Aβ serum level in the LPS-induced group when compared with the control group (I) (DeMattos et al. 2002). In accordance with our results, the disturbance in Aβ metabolism associated with increased Aβ serum triggers a series of immune responses including cytokines and inflammatory factors release (Jensen and Willis 2016; Zuccalà et al. 2005a; Mizrahi et al. 2008). Additionally, following the observed increase in Aβ serum level, a relevant increase in cholesterol and triglycerides serum levels has been also observed as illustrated in Fig. 10. Accordingly, Aβ serum levels were found to be directly proportional to both cholesterol and triglycerides levels contributing to cardiac dysfunction and fatty liver injuries (Yeung et al. 2014; Fioranelli et al. 2018; Anwar et al. 2019a, 2018). In evidence by our results, the increased Aβ, cholesterol, and triglycerides levels resulted in aggressive inflammatory cascades activation and cytokines release including TNF-α, caspase-3, and IL-6 as illustrated in Fig. 10 (Verdile et al. 2015; Anwar et al. 2018, 2019a). Thereby, the observed triggered inflammatory cytokines, and the elevated cholesterol-triglycerides levels are suggested to be mainly derived from the increased Aβ serum level resulted in liver necrosis and cardiac dysfunction as illustrated in Fig. 10 (Stakos et al. 2020; DeMattos et al. 2002; Boada et al. 2009).

As a consequence of the previously mentioned successive inflammatory cascades related to elevated Aβ serum level, cardiac dysfunction and liver necrosis have been observed in AD-induced group when compared with the control group (I). Concerning liver necrosis biomarkers associated with AD progression, a decreased albumin level along with increased ammonia, ALP, AST, and ALT were observed as illustrated in Fig. 10 (Zuccalà et al. 2005a; Mizrahi et al. 2008; Llewellyn et al. 2010b). The suggested inverse association between serum albumin level and Aβ levels in serum and brain tissue directly results in the impaired sink hypothesis and may act as the main triggering factor for liver injury, cardiac dysfunction, and AD progression as illustrated in Fig. 10 (Zuccalà et al. 2005a; Mizrahi et al. 2008; Llewellyn et al. 2010b). Normally, the sink hypothesis suggests that Aβ protein shifts from the brain into the blood circulation forming an observed decrease in Aβ concentration gradient level between the brain and blood level. In this hypothesis, normal serum albumin bind to most Aβ serum level, and in turn lowers Aβ serum concentration. Therefore, normal serum albumin plays a vital role in balancing the dynamic equilibrium of Aβ level between the brain and peripheral circulation (Boada et al. 2009; DeMattos et al. 2002). Intriguingly, low serum albumin mainly results in the less binding capacity to Aβ in the blood resulting in increased Aβ serum level associated with increased Aβ deposition in the brain resulting in severe complications whether in the brain or even in the heart and liver (Cattin et al. 1997a; Kalantar-Zadeh et al. 2004; McCusker et al. 2001; Silverberg et al. 2001). Furthermore, serum albumin constitutes the main barrier system by trapping ROS, NO, and inflammatory cytokines (Roche et al. 2008). In accordance with our results, decreased serum albumin level may results in several complications ending with liver necrosis and cardiac dysfunction (Zuccalà et al. 2005a; Silverberg et al. 2001; Cattin et al. 1997a). Additionally, albumin inhibits Aβ aggregations in brain tissue (Milojevic et al. 2007). Therefore lowered serum albumin may act as a drawback of AD progression (Llewellyn et al. 2010a) and may also lead to increased neuroinflammatory AD type pathology as illustrated in Fig. 10 (Galeazzi et al. 2002).

According to our observed results, decreased albumin level in LPS-induced group was followed by increased ammonia, ALP, AST, and ALT level when compared with the control group (I). In accordance with our results, decreased serum albumin as a drawback of neuroinflammatory AD-type progression may results in server liver dysfunctions due to systemic malnutrition inflammatory syndrome (Kaji et al. 2021; Llewellyn et al. 2010a; Cattin et al. 1997a). Meanwhile, increased Aβ serum level associated with increased cholesterol, triglycerides, and inflammatory cytokines serum levels in addition to decreased albumin level directly resulted in cardiac dysfunction illustrated in the form of increased heart rate, troponin I, CK-MB, LDH, ALT, and AST serum levels as illustrated in Fig. 10. In a line with our results, (Stakos et al. 2020; Cermakova et al. 2015; Yeung et al. 2014; Fioranelli et al. 2018; Murphy et al. 2022; Ewid et al. 2020; Zhu et al. 2022; Shen et al. 2015) indicated that elevated Aβ serum level may be found to be associated with severe hypoxia, low cardiac output, and myocardial dysfunctions leading to heart failure in the long term.

Nowadays, two valid drug types including memantine and cholinesterase inhibitors as a treatment for AD, are clinically proven to improve memory decline and alertness without hindering AD progression and life expectancy (Weller and Budson 2018). No attention has been given to eliminating the drawbacks of AD progression on other organs, therefore, the quality of life of AD patients is consistently kept on decreasing with an increased ratio of mortality and morbidity rate. Thereby the main aim of our current therapeutic protocol was to hinder AD-associated damages on other organs including the liver and heart. Our previous results indicated the efficiency of exogenous hydrogen sulphide donor and MSCs (whether systemic or localized administration) in hindering AD progression and even may act as a successful prophylactic protocol due to their anti-inflammatory, antioxidant, immunomodulating response, and Aβ engulfing actions due to the activation of Microglia M2 anti-inflammatory type over M1 microglia proinflammatory type (Anwar et al. 2018, 2019b, 2021b, 2023; Anwar 2019). Our results revealed that the administration of NaHS as an exogenous hydrogen sulphide donor and MSCs alleviated neuroinflammatory AD damageable drawbacks on the liver and heart by decreasing Aβ, cholesterol, triglycerides, and, inflammatory cytokines in serum levels. Thereby by, a significant improvement has been observed regarding albumin, ammonia, ALP, AST, ALT, HR, troponin I, LDH, and CK-MB serum levels following their administration with a more relevant improvement has been observed following NaHS administration when compared with the control group (I). In line with our observed results, (Ertugrul et al. 2021; Guo et al. 2020; Yang et al. 2021; Sun et al. 2021; Mateus and Prip-Buus 2022) proved the efficiency of the administration of exogenous hydrogen sulphide donor and MSCs in alleviating cardiac and liver dysfunctions by promoting tissue regeneration, inhibiting fibrosis, and regulating immune responses.

## Conclusion

In conclusion, LPS-induced neuroinflammatory rats exhibit impaired cardiac and hepatic functionality when compared with control subjects. Liver and cardiac impairments were demonstrated by histopathological and special staining findings in correlation with observed disturbance in cardiac and hepatic normal biomarkers. Overall, these results are mainly proposed to be related to increased Aβ serum level triggering serious of inflammatory cascades, immune responses, and oxidative stress, The mechanisms responsible for exhibiting Aβ-related cardiac and hepatic dysfunctions was clearly identified and proven along with providing a clue to combat neuroinflammation drawbacks on other organs in order to improve the quality of life of AD patients by suggesting a dual way of therapeutic interventions. Our suggested therapeutic approach included the administration of NaHS and MSCs which proved their efficiency in alleviating cardiac and hepatic dysfunctions by hindering the extent of increased Aβ serum level drawbacks and thereby restoring normal heart and liver functions. Based on our observations and other reasonable evidence supporting the association between AD and hepatic-cardiac dysfunctions, the current study advocates for a major shift toward a greater interest in providing various types of successful therapeutic interventions for combating AD drawbacks whether on the brain or on other organs in order to hinder the overall neuroinflammatory AD status progression.

## Data Availability

The obtained data analyzed during the current study are available from the corresponding author on reasonable request.

## Abbreviations

AD: Alzheimer’s disease
Aβ: Amyloid beta
BBB: Blood–brain barrier
LPS: Lipopolysaccharide
PBS: Phosphate buffer saline
NaHS: Sodium hydrosulfide
MSCs: Mesenchymal stem cells
APP: Amyloid precursor protein
CVD: Cerebrovascular disease
ALT: Alanine aminotransferase
AST: Aspartate aminotransferase
ALP: Alkaline phosphatase
PBS: Phosphate-buffered saline
DMEM: Dulbecco's modified Eagle's medium
FBS: Fetal bovine serum
MWM: Morris water maze
LDH: Lactate dehydrogenase
CK-MB: Creatine kinase-MB
TNF-α: Tumor necrosis factor-alpha
TP: Total protein
IL-6: Interleukin 6
